# Microwave-Assisted Pillaring of a Montmorillonite with Al-Polycations in Concentrated Media

**DOI:** 10.3390/ma10080886

**Published:** 2017-08-01

**Authors:** Beatriz González, Alba Helena Pérez, Raquel Trujillano, Antonio Gil, Miguel A. Vicente

**Affiliations:** 1 Departamento de Química Inorgánica, Universidad de Salamanca, 37008 Salamanca, Spain; bei@usal.es (B.G.); ahpj@usal.es (A.H.P.); rakel@usal.es (R.T.); 2 Departamento de Química Aplicada, Universidad Pública de Navarra, 31006 Pamplona, Spain; andoni@unavarra.es

**Keywords:** Al-PILC, Keggin polycation, concentrated media, microwave radiation, pillared montmorillonite

## Abstract

A montmorillonite has been intercalated with Al^3+^ polycations, using concentrated solutions and clay mineral dispersions. The reaction has been assisted by microwave radiation, yielding new intercalated solids and leading to Al-pillared solids after their calcination at 500 °C. The solids were characterized by elemental chemical analysis, X-ray diffraction, FTIR spectroscopy, thermal analyses, and nitrogen adsorption. The evolution of the properties of the materials was discussed as a function of the preparation conditions. Microwave treatment for 2.5 min provided correctly pillared solids.

## 1. Introduction

Clay minerals’ importance in the field of catalysis is booming, because they have high specific surface area and active acid centers that lead to their use in various reactions such as gasoline desulfurization, terpene isomerization, olefin polymerization, cracking, and numerous fine chemical reactions [1].

One of the most studied groups of clay minerals is that of smectites, which are 2:1 phyllosilicates. The expansibility of the smectite interlayer space depends on four fundamental factors: the nature of the exchangeable, charge-compensating cations, the density of the surface charge and the location of the charge. The best known smectite is montmorillonite, very abundant in nature and with a great variety of properties and applications. Montmorillonites are formed by silicates of Al, Mg, or Fe with various degrees of hydration and amounts of alkaline and alkaline earth exchangeable cations, with a cation exchange capacity (CEC) between 0.3 and 0.8 meq/g [2]. Their basal space ranges from 9.7 Å when the sheets are as close as possible, to 12–14 Å in natural samples, when the interlayer cations are hydrated by a monolayer or a bilayer of water molecules, and to about 23 Å when intercalating voluminous cations. Montmorillonite is one of the clay minerals with higher industrial interest, being easy to obtain at low cost.

Pillared clays (PILC) are a family of molecular engineered porous solids very studied in the last decades. Its preparation is based on the exchange of interlayer cations of the natural clay mineral by voluminous inorganic polycations, obtained by polymerization of various multivalent cations (Al^3+^, Ga^3+^, Ti^4+^, Zr^4+^, Fe^3+^, among others). The resulting solids are calcined at moderate temperatures, up to 500 °C, leading to stable materials with a larger spacing than in the initial solid. During calcination, polycations are converted into metal oxides, pillars, inserted between the sheets of the clay mineral and keeping them separated from each other, which prevents the collapse of the structure [3,4,5,6]. The most commonly used polycation in the pillaring process is the Keggin polycation, [Al_13_O_4_(OH)_24_(H_2_O)_12_]^7+^, due to its stability and simple preparation [7,8,9].

In addition to the nature of the polycation, there are other factors that condition the intercalation process and that must be considered when planning a preparation, such as intercalation time, hydrolysis conditions, and aging of the polycations. Usually, dilute solutions of the polycations and dilute dispersions of the clay minerals are used, looking for optimal conditions for the polymerization of the cations and for the cation exchange reaction required for substituting the compensating cations of the clay mineral by the polycations. Logically, this involves the use of large volumes of water, strongly hindering the scaling-up of this process to prepare large amounts of pillared solids. Some papers have proposed procedures to solve this drawback; these papers are reviewed in [6].

Microwave-hydrothermal treatment has been widely used in recent years in materials science, making the preparation reactions faster than using traditional methods. In clay science, microwave-assisted methods have been used for the preparation of clay minerals, mainly saponite [10], and of Layered Double Hydroxides (LDH) [11], and also for the preparation of PILC [6,12].

The aim of this work is to obtain montmorillonites pillared with aluminum Keggin polycations using microwave radiation and concentrated media—that is, low water volumes—as well as to determine the structural and textural properties of the solids thus obtained.

## 2. Materials and Methods

### 2.1. Preparation of the Solids

The clay mineral used was a raw montmorillonite from Cheto, Arizona, USA (from The Clay Minerals Repository, where this sample is denoted as SAz-1). The natural clay mineral was purified before its use by dispersion–decantation, separating the fraction lower than 2 μm. In the present work, this clay mineral is designated as ‘RMt’. Its cation exchange capacity was 0.67 meq/g, its basal spacing was 13.60 Å, and its BET specific surface area was 49 m^2^/g [13]. For comparison, this solid was calcined at 500 °C, the same temperature as the pillared solids, denoted as ‘Mt’.

Four samples were synthesized, varying the aluminum concentration and the treatment time under microwave irradiation. In a typical experiment, the Al-polycation solution was prepared by dissolution of 10 mmol of AlCl_3_·6H_2_O in 20 cm^3^ of water and the subsequent slow addition, under vigorous stirring of 22 mmol of NaOH until pH = 4.2 was reached, the condition for the formation of Al_13_^7+^ polycations. This intercalating solution was added dropwise to a previously prepared montmorillonite dispersion (2 g in 20 cm^3^), with an Al/montmorillonite ratio of 5 mmol/g, and it was submitted to microwave treatment at 150 °C several times. For that, the suspensions were sealed in 100 cm^3^ Teflon reactors and treated in a Milestone Ethos Plus microwave furnace. The heating process was programmed with EasyWAVE Software. The furnace has a power of 600 W, and initially applies the power needed for a heating ramp of 5 °C/min to the treatment temperature and then to maintain the temperature for the time required. Then, the solids were separated and washed by centrifugation, dried overnight at 70 °C, and finally calcined at 500 °C for 2 h, with a heating rate of 1 °C/min. The conditions of preparation and the names of the solids are summarized in Table 1. All reagents used were supplied by Panreac (Barcelona, Spain), being compounds of the highest purity, and were used without purification.

### 2.2. Characterization Techniques

Element chemical analyses were carried out at *Servicio General de Análisis Químico Aplicado* (University of Salamanca, Salamanca, Spain), using Inductively Coupled Plasma-Atomic Emission Spectrometry (ICP-AES). X-ray diffraction (XRD) patterns were recorded between 2° and 65° (2θ) over non-oriented powder samples, at a scanning speed of 2° (2θ)/min, by using a Siemens D-500 diffractometer (Siemens España, Madrid, Spain), operating at 40 kV and 30 mA, using filtered Cu Kα radiation (λ = 1.5418 Å). FT-IR spectra were recorded between 450 and 4000 cm^−^^1^ using a PerkinElmer Spectrum-One spectrometer (Waltham, MA, USA) by the KBr pellet method, with a sample:KBr ratio of 1:300. Thermal analyses were performed on a SDT Q600 TA instrument (New Castle, PA, USA), TG and DTA were carried out simultaneously; all measurements were carried out under a flow of 20 cm^3^/min of oxygen (Air Liquide, Madrid, Spain, 99.999%) and a temperature heating rate of 10 °C/min from room temperature to 900 °C. Textural properties were determined from nitrogen (Air Liquide, 99.999%) adsorption–desorption data, obtained at −196 °C using a Micrometrics Gemini VII 2390t (Norcross, GA, USA), Surface Area and Porosity apparatus. Specific surface area (SSA) was obtained by the BET method, external surface area and micropore volume by means of the *t*-method, and the total pore volume from the nitrogen adsorbed at a relative pressure of 0.95 [14]. 

## 3. Results and Discussion

Characterization or raw montmorillonite has been reported elsewhere [13], it is a very pure clay mineral.

The elemental chemical compositions of all the Al-PILC samples are given in Table 2. For better assessment of the chemical effects of the treatments, a double normalization was carried out. First, the composition was given as for water-free solids, that is, the metal oxide content was normalized to sum 100%, the effect of the content of water in each solid was thus avoided. Then, the compositions were referred to the content of SiO_2_ in the original Mt, as the tetrahedral sheet of the montmorillonite was not expected to be affected by the intercalation and pillaring treatment, the amount of SiO_2_ thus remaining constant, and it can be used as an ‘internal standard’. The compositions thus normalized are given in Table 3.

The amount of aluminum fixed, once the amount existing in the raw montmorillonite was subtracted, was very similar in all the solids, between 14.58% and 16.60% (expressed as Al_2_O_3_), enough to intercalate the montmorillonite compensating its CEC. In fact, the polycations were incorporated into the clay mineral structure by cationic exchange of Ca^2+^, its main exchangeable cation, which was almost completely removed, while K^+^ content remained almost constant, suggesting that it was in the raw montmorillonite as feldspar, but not as exchangeable cations. The amount of Fe_2_O_3_ remained essentially constant, while MgO slightly decreased, suggesting that octahedral Mg^2+^ was scarcely dissolved. The polymerization of Al^3+^ required relatively acid conditions (pH = 4.2), but the contact of the clay mineral with the polycation solution did not significantly alter the composition of the Mt layers.

**Figure 1:**
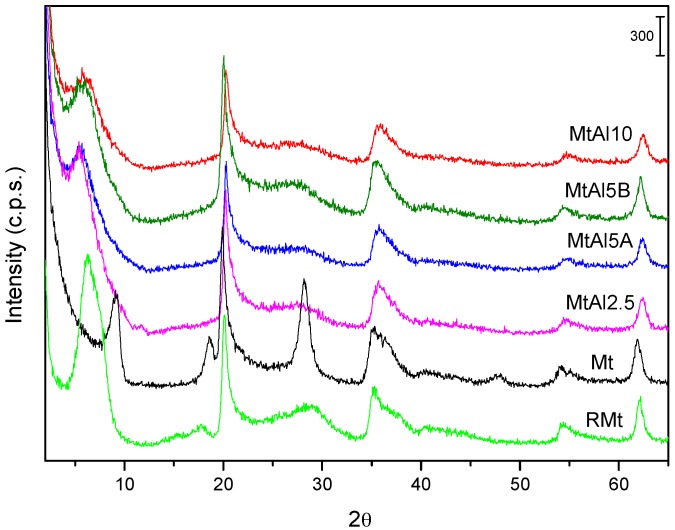
Powder X-ray diffractograms of the pillared solids calcined at 500 °C. For comparison, the diffractograms of the raw montmorillonite (RMt) and of this sample calcined at 500 °C (Mt) are also given.

The powder X-ray diffractograms of pillared solids are shown in Figure 1 and the basal spacing of all the solids are summarized in Table 4. The raw solid was a well-ordered Mt with a basal spacing of 13.90 Å, which collapsed to 9.71 Å after calcination at 500 °C [13]. The treatment with the intercalating solutions always led to the expansion of the interlayer region, the basal spacing varying between 15.87 Å and 18.14 Å for the intercalated, dried solids (Table 4). The maximum value, 18.14 Å, similar to the values usually reported under conventional pillaring, was observed for MtAl5A solid, implying a height of the interlayer region of approximately 8.4 Å, which coincided with the height of the Al_13_^7+^ polycations. The other three solids did not reach this value, remaining around 16 Å, which supposes a height of the interlayer region of ~6.3 Å, suggesting that the polymerization was not optimal or that the previously formed polycations suffered some changes in the degree of polymerization during their addition to the clay mineral or during the microwave treatment.

In general, the basal spacing decreased during the calcination process. Calcination of intercalated samples usually produces a decrease in their basal spacing by dehydration and dehydroxylation of the Al_13_^7+^ polycations until the formation of clusters close to Al_2_O_3_, and this is the trend found for MtAl5A, MtAl5B, and MtAl10 solids. However, in the case of the MtAl2.5, there was an increase in the basal spacing during calcination. This sample had been submitted to a lower time of treatment with microwave radiation, so it seems that the intercalation had not been completed during this treatment and the process could be completed at the beginning of the calcination. 

The effects due to in-layer reflections, independent of *c*-stacking, were recorded at the same positions for all the solids, indicating that the layers were not modified. No reflections belonging to crystalline Al_2_O_3_ were observed.

The FWHM (Full Width at Half Maximum) index of the basal 001 reflection varied from the intercalated to the calcined solids (Table 5), showing changes in the long-distance arrangement according to this plane, i.e., the number of layers correctly stacked along the *c* axis. Raw montmorillonite had a relatively small FWHM index, probably due to the ordering caused by the dispersion–decantation purification procedure (when calcining at 500 °C, a very sharp peak, with FWHM of only 0.999° was obtained, due to the collapse to TOT arrangement). The intercalation tended to order the sheets, that is, the interaction with the polycations made more layers to correctly stack, due to the successive interaction layers–polycations. Calcination for forming the final pillared solids increased or decreased the value of FWHM index, depending of the considered solid. Usually, calcination causes a decrease of ordering, and this actually occurred in MtAl5B and MtAl10, however, the opposite behavior was observed for MtAl2.5 and MtAl5A. This may be due to the high speed of the intercalation procedure in these solids, which may not allow the correct stacking of the layers, by which the stacking may continue during the first steps of the calcination. 

**Figure 2:**
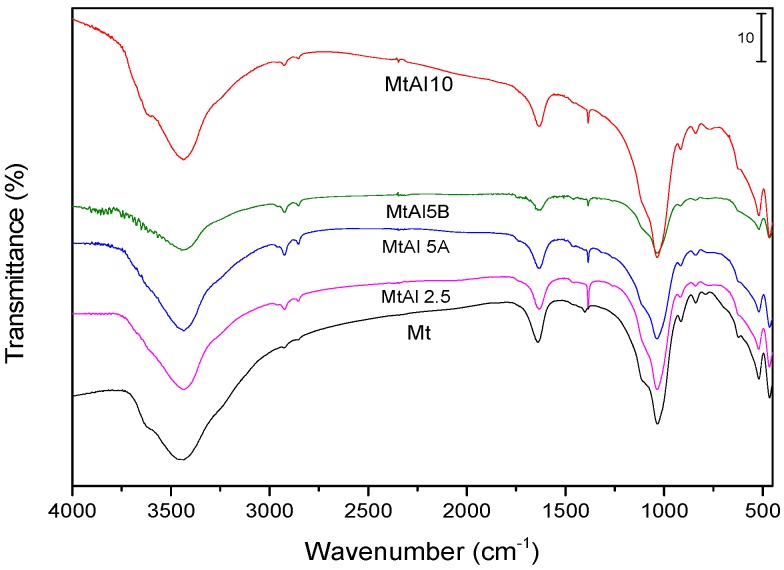
FT-IR spectra of the intercalated solids.

The FT-IR spectra of the all samples were similar to that of natural Mt (Figure 2). The O–H stretching band was recorded close to 3440 cm^−1^ and the H–O–H bending vibrational mode at 1637 cm^−1^. Bands corresponding to the Si–O–Si and Si–O–Al vibrations were recorded at 1036 and 522 cm^−1^. No Al–O bands were observed for the intercalated clays. The intensity of the O–H stretching mode of the hydroxyls bonded to the metals, recorded around 3600 cm^−1^, increased after intercalation in MtAl10, suggesting an interaction with the polycations, and an increase in the acidity of this solid. 

**Figure 3:**
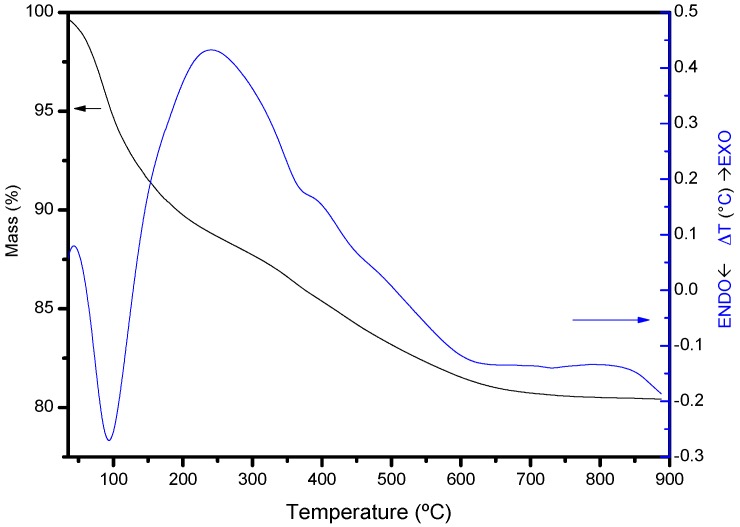
Thermal curves, TG, and DTA of MtAl10 solid.

The thermogravimetric curve of the intercalated solids (that from MtAl10 is included in Figure 3 as an example) showed a ~12% mass loss at low temperature, associated with an endothermic effect centered at 94 °C in the DTA curve. This effect was due to the removal of water located in the interlayer region, bonded to the polycations, or adsorbed on the external surface of the sheets. In the raw montmorillonite, this effect produced an 11% mass loss, with an endothermic associated effect centered at 100 °C. Thus, the loss of the exchangeable cations and the cations coordinated to them was compensated by the water adsorbed on the Al_13_^7+^ polycations, giving up to similar effects for both solids. In the central temperature range, a significant mass loss effect (~6%) was observed in the pillared solids, with three slight inflexions, associated with an exothermic effect centered at 242 °C and a shoulder at 396 °C. These effects are not observed in the raw montmorillonite, and should be due to the dehydration of the polycations, confirming their correct intercalation on montmorillonite interlayer.

**Figure 4:**
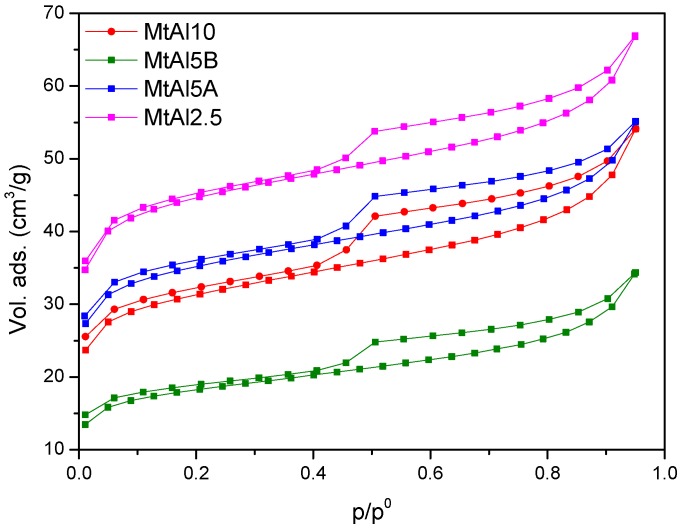
Nitrogen adsorption–desorption isotherms of the pillared solids.

The textural properties were studied by N_2_ adsorption–desorption experiments. The adsorption isotherms (Figure 4) belonged to type II from IUPAC classification, with a H4 type hysteresis loop at high relative pressures, a form associated with narrow slit pores [15]. The loop had an inflexion at a relative pressure value of 0.4, being reversible at low p/p° values for the non-calcined solids, indicating that pores were not rigid but flexible, and became practically reversible for the calcined solids.

BET specific surface area, external surface area, and micropore volume data are summarized in Table 6. Intercalated solids had specific surface areas higher than the starting clay mineral, with values between 107 and 137 m^2^/g. The intercalation of the Al_13_^7+^ polycations produced a remarkable separation of the sheets, which would tend to increase the specific surface; however the interlayer region was occupied by the polycations, so the nitrogen molecules could not freely access it. Both effects compensated among them, but the first was predominant. The porosity ranged between 0.039 and 0.054 cm^3^/g and the external surface was in the range of 36–45 m^2^/g (~28–37% of the total surface area).

Calcination produced a decrease in the S_BET_ for MtAl5A, MtAl5B, and MtAl10 solids, which aligned with the expected trend, a decrease of this magnitude due to the collapse of the sheets, while the external surface remained practically constant, increasing its relative contribution to the total surface, up to 47%. Again, MtAl2.5 showed opposite behavior, with the S_BET_ increasing upon calcination. As previously commented, this was the solid submitted to a shorter time of microwave treatment. The increase in surface area again suggested that the pillaring procedure was completed during the calcination step. 

The overall analysis of these results indicated that Al-PILC can be obtained by microwave treatment of concentrated solutions and dispersions for a time as short as 2.5 min, although in this case, the pillaring process seemed to be completed during the final calcination step of the preparation procedure. Thus, microwave radiation should strongly facilitate the preparation of these solids in large amounts.

## 4. Conclusions

Montmorillonite was effectively pillared with Al_13_^7+^ polycations, using microwave radiation and small volumes of concentrated solutions of the polycations and concentrated dispersions of the clay mineral. The solids obtained showed structural characteristics comparable to Al-PILC prepared from the classical method. Thus, the use of microwave radiation allows a significant reduction in the times of treatment and the volumes of solutions/dispersions required by the conventional preparation method.

## Figures and Tables

**Table 1:** Nomenclature of the solids and preparation conditions.

Name	Dispersion Concentration (g/cm^3^)	Intercalating Solution Concentration (mmol Al^3+^/cm^3^)	Microwave Treatment Time (min)
MtAl2.5	2/20	10/20	2.5
MtAl5A	2/20	10/20	5
MtAl5B	2/10	10/10	5
MtAl10	2/20	10/20	10

**Table 2:** Chemical composition of the solids, expressed in content of their metallic oxides (mass percentage).

Sample	SiO_2_	Al_2_O_3_	Fe_2_O_3_	MnO	MgO	CaO	Na_2_O	K_2_O	TiO_2_	Total
RMt	55.80	15.92	1.41	0.04	5.58	1.69	0.06	0.06	0.21	80.77
MtAl2.5	49.11	24.37	1.21	0.02	4.10	0.05	0.35	0.13	0.18	79.52
MtAl5A	53.76	28.25	1.35	0.02	5.21	0.04	0.37	0.12	0.20	89.32
MtAl5B	55.45	28.04	1.37	0.03	4.96	0.03	0.36	0.04	0.20	90.48
MtAl10	53.89	28.13	1.28	0.03	4.96	0.04	0.35	0.10	0.19	88.97

**Table 3:** Chemical composition of the water-free solids, normalized to the SiO_2_ content in the raw montmorillonite (mass percentage).

Sample	SiO_2_	Al_2_O_3_	Fe_2_O_3_	MnO	MgO	CaO	Na_2_O	K_2_O	TiO_2_
Mt	69.09	19.71	1.75	0.05	6.91	2.09	0.07	0.07	0.26
MtAl2.5	69.09	34.29	1.70	0.03	5.77	0.07	0.49	0.18	0.26
MtAl5A	69.09	36.31	1.73	0.02	6.69	0.05	0.47	0.15	0.25
MtAl5B	69.09	34.94	1.70	0.03	6.18	0.03	0.45	0.05	0.25
MtAl10	69.09	36.07	1.64	0.03	6.35	0.05	0.44	0.13	0.24

**Table 4:** Basal spacing (Å) for intercalated and calcined samples.

Sample	Intercalated	500 °C
Mt	13.90 *	9.71
MtAl2.5	16.14	16.50
MtAl5A	18.14	15.58
MtAl5B	15.87	15.56
MtAl10	16.22	15.58

* For Mt sample, basal spacing of the raw montmorillonite.

**Table 5:** Value of FWHM index of the solids.

Solid	FWHM/°
RMt	2.372
Mt-500	0.999
MtAl2.5	1.929
MtAl2.5-500	1.775
MtAl5A	1.768
MtAl5A-500	1.701
MtAl5B	1.701
MtAl5B-500	2.290
MtAl10	1.607
MtAl10-500	1.887

**Table 6:** Specific surface area (S_BET_), external surface area (S_ext_) and micropore volume (V_m_) of natural montmorillonite [13] and of the pillared solids.

Sample	S_BET_ (m^2^/g)	S_t_ (m^2^/g) *	V_m_ (cm^3^/g)
RMt	49	49 (100)	0.000
Mt-500	80	64 (80)	0.009
MtAl2.5	123	45 (37)	0.044
MtAl2.5-500	135	49 (36)	0.047
MtAl5A	137	39 (28)	0.054
MtAl5A-500	107	43 (40)	0.035
MtAl5B	107	37 (35)	0.039
MtAl5B-500	57	27 (47)	0.016
MtAl10	115	36 (31)	0.044
MtAl10-500	96	43 (45)	0.030

* In brackets, percentage of external surface to the total specific surface area.
